# Genomic Aberrations in Circulating Tumor DNAs from Palbociclib-Treated Metastatic Breast Cancer Patients Reveal a Novel Resistance Mechanism

**DOI:** 10.3390/cancers14122872

**Published:** 2022-06-10

**Authors:** Maysa Abu-Khalaf, Chun Wang, Zhenchao Zhang, Rui Luo, Weelic Chong, Daniel P. Silver, Frederick Fellin, Rebecca Jaslow, AnaMaria Lopez, Terrence Cescon, Wei Jiang, Ronald Myers, Qiang Wei, Bingshan Li, Massimo Cristofanilli, Hushan Yang

**Affiliations:** 1 Department of Medical Oncology, Sidney Kimmel Cancer Center, Thomas Jefferson University, Philadelphia, PA 19107, USA; chun.wang@jefferson.edu (C.W.); zhenchao.zhang@jefferson.edu (Z.Z.); rui.luo@jefferson.edu (R.L.); weelic.chong@students.jefferson.edu (W.C.); daniel.silver@jefferson.edu (D.P.S.); frederick.fellin@jefferson.edu (F.F.); rebecca.jaslow@jefferson.edu (R.J.); anamaria.lopez@jefferson.edu (A.L.); ronald.myers@jefferson.edu (R.M.); 2 Department of Hematology Oncology, Reading Hospital, West Reading, PA 19611, USA; terrence.cescon@towerhealth.org; 3 Department of Pathology, Sidney Kimmel Cancer Center, Thomas Jefferson University, Philadelphia, PA 19107, USA; wei.jiang@jefferson.edu; 4 Department of Molecular Physiology and Biophysics, Vanderbilt University, Nashville, TN 37235, USA; qiang.wei@vanderbilt.edu (Q.W.); bingshan.li@vanderbilt.edu (B.L.); 5 Division of Hematology and Medical Oncology, Department of Medicine, Weill Cornell Medicine, New York, NY 10021, USA; mac9795@med.cornell.edu

**Keywords:** circulating tumor DNA, somatic mutation, metastatic breast cancer, palbociclib, treatment resistance

## Abstract

**Simple Summary:**

The combination of CDK4/6 inhibitors and endocrine therapy is now considered the standard of care for patients with estrogen receptor-positive (ER^+^) breast cancer. The emerging tide of CDK4/6 inhibitor resistance urges more research to explore underlying resistance mechanisms. This study profiled circulating cell-free DNA obtained before and after palbociclib treatment in ER^+^ metastatic breast cancer patients using 91-gene panel sequencing. The results revealed that the acquisition of *CCNE1* mutations and the loss of *TSC2* mutations confer an unfavorable prognosis, suggesting that these mutations may potentially serve as novel genomic biomarkers for treatment resistance. Future large-scale population studies and mechanism-focused research are needed to confirm the findings of this study and elucidate the underlying molecular mechanisms. Ultimately, such efforts may lead to the development of improved methods to predict treatment efficacy and clinical outcomes, as well as more effective targeted treatment approaches for the benefit of breast cancer patients.

**Abstract:**

Previously undescribed molecular mechanisms of resistance will emerge with the increased use of cyclin-dependent kinase 4/6 inhibitors in clinical settings. To identify genomic aberrations in circulating tumor DNA associated with treatment resistance in palbociclib-treated metastatic breast cancer (MBC) patients, we collected 35 pre- and post-treatment blood samples from 16 patients with estrogen receptor-positive (ER^+^) MBC, including 9 with inflammatory breast cancer (IBC). Circulating cell-free DNAs (cfDNAs) were isolated for sequencing using a targeted panel of 91 genes. Our data showed that *FBXW7* and *CDK6* were more frequently altered in IBC than in non-IBC, whereas conversely, *PIK3CA* was more frequently altered in non-IBC than in IBC. The cfDNA samples collected at follow-up harbored more mutations than baseline samples. By analyzing paired samples, we observed a higher percentage of patients with mutations in *RB1*, *CCNE1*, *FBXW7*, *EZH2*, and *ARID1A*, but a lower proportion of patients with mutated *TSC2* at the post-treatment stage when they developed progression. Moreover, acquisition of *CCNE1* mutations or loss of *TSC2* mutations after treatment initiation conferred an unfavorable prognosis. These data provide insights into the relevance of novel genomic alterations in cfDNA to palbociclib resistance in MBC patients. Future large-scale prospective studies are warranted to confirm our findings.

## 1. Introduction

Breast cancer is the most commonly diagnosed cancer and the leading cause of cancer death in American women [1]. Among the 3.8 million women with a history of breast cancer living in the United States, more than 150,000 women have metastatic disease [2]. The 5-year survival rate is 27% for patients with metastatic breast cancer (MBC), far lower than the survival rate of >90% observed in patients with early-stage disease [2]. The estrogen receptor-positive (ER^+^) breast cancer subtype represents up to 80% of MBC [3]. Although endocrine therapy is the bedrock of adjuvant systemic therapy for patients with early-stage ER^+^ breast cancer and results in improved survival, patients with ER^+^ metastatic disease will eventually exhibit endocrine therapy resistance after the first two lines of treatment, frequently followed by rapid clinical deterioration and a dismal prognosis [4]. Therefore, the development of effective combination therapies targeting this prevalent subtype of MBC will substantially improve patient survival.

The addition of cyclin-dependent kinase 4/6 (CDK4/6) inhibitors to regimens for advanced ER^+^ breast cancer is one of the most significant advances in the last decade [5,6]. Clinical trials have demonstrated that palbociclib significantly increases the progression-free survival (PFS) of patients with advanced ER^+^ and HER2-negative (HER2^−^) breast cancer, not only in the first-line setting in combination with letrozole, but also in the second-line setting in combination with fulvestrant after disease progression following endocrine therapy [7,8,9]. In 2015, palbociclib became the first CDK4/6 inhibitor approved by the Food and Drug Administration (FDA) for the treatment of ER^+^/HER2^−^ MBC. Since then, the FDA approved two additional CDK4/6 inhibitors and the combination of CDK4/6 inhibitors and endocrine therapy in the first- or second-line setting is now considered the standard of care for advanced ER^+^ breast carcinoma [6]; however, the development of resistance to CDK4/6 inhibitors is inevitable in patients undergoing this treatment. In the clinical trials that led to the FDA’s approval, at least 1/3 of patients relapsed on CDK4/6 inhibitors within 2 years, and in the PALOMA-2 trial, >70% of patients treated with the palbociclib plus letrozole combination had progressive disease within 40 months [5,8]. Therefore, the identification of predictive and prognostic molecular biomarkers associated with resistance to CDK4/6 inhibitors is an emerging field of interest in the cancer research community.

In breast cancer, the deregulation of key players in the cyclin D-CDK4/6-Rb signaling cascade promotes unchecked cell proliferation [5,10]. CDK4/6 inhibitors are small-molecule kinase inhibitors that competitively occupy the ATP-binding pocket of CDK4 and CDK6, preventing the phosphorylation of Rb, and thus leading to cell cycle arrest [10]. Preclinical studies have identified a number of putative mechanisms of resistance to CDK4/6 inhibitors, including the loss or acquisition of *RB* mutations, altered CDK4/6 and CDK2 signaling, and activation of growth signaling pathways [5,11]. Evidence from preclinical settings was corroborated by recent clinical evidence. For example, based on the PALOMA-3 trial, O’Leary et al. [11] detected *RB1* mutations in the plasma of ER^+^/HER2^−^ advanced breast cancer patients at the end of treatment, suggesting the acquisition or selection of genomic aberrations under pressure from palbociclib. Nonetheless, since the incidence of *RB* gene deletion/mutation is rare in ER^+^ breast cancer [5,11,12], it thus does not explain the high prevalence of disease recurrence on CDK4/6 inhibitors. Moreover, it is likely that previously undescribed molecular mechanisms of resistance will emerge with the increased use of CDK4/6 inhibitors in clinical settings. A better understanding of resistance mechanisms is essential for the development of optimal management strategies when breast cancer patients are treated using CDK4/6 inhibitors.

Liquid biopsy is an emerging innovation in precision oncology due to its ability to provide non-invasive, reproducible, and real-time monitoring of cancer status and treatment response [13,14]. Circulating tumor DNA (ctDNA) is one of the most promising liquid biopsy methods. ctDNA comprises a small portion of total circulating cell-free DNA (cfDNA), which is composed of both ctDNAs and DNAs that are derived from normal cells. ctDNA liquid biopsy has been studied most extensively in patients with established metastatic disease and has taken hold in some routine clinical applications, focused mostly on identifying specific therapeuthically actionable tumor alterations [15]. An approach for identifying and characterizing acquired drug resistance using ctDNA has also emerged. In this study, using paired plasma samples collected before and after palbociclib treatment, we were able to identify genomic aberrations in ctDNA that were associated with treatment response and disease progression in MBC patients receiving palbociclib.

## 2. Materials and Methods

### 2.1. Study Population

Female MBC patients with ER^+^ tumors were identified from an ongoing prospective breast cancer cohort established at the Sidney Kimmel Cancer Center, Thomas Jefferson University Hospital [16]. An MBC diagnosis was based on histology confirmation and radiological evaluation. The patients included in this study were those who (1) received palbociclib (single agent or in combination with other agents); (2) had blood samples collected at baseline before treatment; (3) had at least one blood sample collection during follow-up; (4) had radiological restaging evaluations of metastatic disease during follow-up. Demographic and clinical information was obtained by reviewing medical charts. Tumor response was re-assessed during follow-up as standard of care by imaging-based methods, complemented by histological examinations when needed. Treatment response and outcome were evaluated according to the Response Evaluation Criteria in Solid Tumors (RECIST) guideline [17]. The study was approved by the Institutional Review Board of Thomas Jefferson University. An informed consent was signed by each patient for participating in this study.

### 2.2. Plasma Separation

Approximately 10 mL of whole blood was collected at each visit using an EDTA tube. Within 2 h of the blood draw, the blood samples were first centrifuged at 1700× *g* for 10 min at room temperature to separate cells from plasma. The supernatant was then centrifuged at 20,000× *g* for 10 min at 4 °C to remove cell debris. Plasma aliquots were stored at −80 °C prior to DNA extraction. 

### 2.3. DNA Extraction

Before using plasma samples for circulating nucleic acid extraction, tubes were thawed at room temperature and centrifuged at 16,000× *g* for 5 min at 4 °C to remove cryoprecipitates. cfDNAs were isolated, purified, and concentrated from 1.5 mL of plasma using the QIAamp Circulating Nucleic Acid Kit (Qiagen, Hilden, Germany). After extraction, cfDNA was quantified using the Qubit dsDNA HS Assay Kit in the Qubit 2.0 Fluorometer (Invitrogen, Carlsbad, CA, USA).

### 2.4. Targeted Gene Panel Sequencing

cfDNAs were assayed by capture-based next-generation sequencing using an in-house developed panel that targets the complete coding regions of 91 genes with known relevance to breast cancer (Table S1). DNA libraries were prepared from 20 ng or more of plasma DNA input per sample using the QIAseqTM Targeted DNA Panel Kit (Qiagen, Hilden, Germany). Purified products were subjected to strict quality control, including measurement of DNA amounts using the Qubit dsDNA assay and assessment of DNA concentration, integrity, and distribution by the Tapestation or Bioanalyzer (Agilent, Santa Clara, USA). Unique molecular index (UMI)-based sequencing was performed on the Illumina HiSeq platform to generate paired-end 150 bp sequencing reads. For quality control, negative control samples (ddH_2_O) were processed and sequenced with plasma cfDNA samples.

### 2.5. Somatic Mutation Calling

Sequencing data analysis and mutation identification were conducted using an in-house developed computational pipeline [18]. In brief, raw data were trimmed by Fastp v0.20.0 to remove adapter sequences and low-quality reads, and then clean data were aligned to the reference human genome (hg19) using the Burrows–Wheeler Aligner (BWA) v0.7.17 [19]. The BAM files were further processed following the best practices [20], including PCR duplicates removed by Picard v1.119 (http://broadinstitute.github.io/picard accessed on 1 April 2022), read pairs realigned around potential indel regions by the Genome Analysis Toolkit (GATK) IndelRealigner v3.6 [21], base quality recalibrated by GATK BaseRecalibrator, and variants filtered by GATK VariantFiltration. ANNOVAR was used to annotate mutations with functional relevance. We applied the following filtering criteria for somatic mutation calling: (1) variants were not reported as germline in the dbSNP database (except for those also reported in COSMIC) with a minor allele frequency <0.0001, or not reported in the Exome Aggregation Consortium project or Genome Aggregation Database [22,23]; (2) at least five reads in each strand supported the alternative allele; (3) variant allele frequency (VAF) was >1%. The analyses in this study focused on functional mutations in exomes, including nonsynonymous SNV, frameshift/non-frameshift insertion and deletion, stop gain, and stop-loss variants. R package maftools was used to visualize the mutational landscape and comprehensively analyze and compare somatic variants in paired samples. The Sankey diagram (R package “ggalluvial”) was used to illustrate changes in individual mutations of each mutated gene from baseline to follow-up.

### 2.6. Statistical Analyses

Frequencies of mutated genes in unpaired samples of two comparison groups were compared using Fisher’s exact test. Frequencies of mutated genes in paired samples collected at baseline and follow-up from the same patients were compared using the McNemar’s test [11]. Median variant counts or VAFs were compared between two groups by the Wilcoxon test. Change in VAFs of a given mutation from baseline to follow-up was evaluated by a paired *t*-test. PFS was defined as the time from baseline blood draw to disease progression or death from any cause. Survival curves were plotted using the Kaplan–Meier method and survival differences between patients who acquired/lost or did not acquire/lose gene mutations were compared using the log-rank test. The swimmer plot (R package “ggplot2”) was used to visualize tumor radiographic responses during follow-up for individual patients. Statistical analyses and result visualizations were carried out by R (version 4.1.0) and GraphPad Prism (version 9, San Diego, CA, USA) software. All *p* values were two-sided, with a *p* < 0.05 considered as statistically significant. 

## 3. Results

### 3.1. Patient Characteristics

Sixteen ER^+^ MBC patients (15 HER2^−^ and 1 HER2^+^) with a median age of 54.7 years old (range 32.6–74.8 years) who received palbociclib for a metastatic breast cancer diagnosis were identified from the cohort of 162 breast cancer patients enrolled in the study between February 2014 to April 2016, which preceded FDA approval for ribociclib and abemaciclib. Among these patients (Table 1), the majority were white (87.5%), had inflammatory breast cancer (IBC, 56.3%), had a poorly differentiated tumor (56.3%), and had previously received at least one line (median: 1.5 lines) of systemic therapy (87.5%). Except for one patient (No. 1) with a HER2^+^ tumor who received palbociclib plus TDM-1, all other patients received palbociclib plus endocrine therapy, including eight that received palbociclib plus fulvestrant, and seven received palbociclib plus letrozole. During a median follow-up of 35.1 weeks (range 9.1–100.1 weeks), 14 (87.5%) patients developed progression. Among the remainder, one was lost to follow-up and another patient’s treatment was changed to a different regimen due to side effects. A total of 35 plasma samples were collected from these patients, including 16 baseline samples collected before therapy initiation, 10 samples at the time of disease progression for patients who developed disease progression during the follow-up phase, and 9 samples from patients who continued to respond to treatment during the study follow-up and at a time when disease progression was not detected (4 at the partial response (PR) stage and 5 at the stable disease (SD) stage) (Table 1). Thus, ten baseline-progressive disease (PD) pairs and nine baseline-nonPD pairs (three baseline samples from patient No. 6, 8, and 16 were used for both baseline-PD and baseline-nonPD pairs for each of these three patients, Table 1) were constructed for paired analyses. 

### 3.2. Genomic Variants in cfDNAs at Baseline and Follow-Up

**Figure 1:**
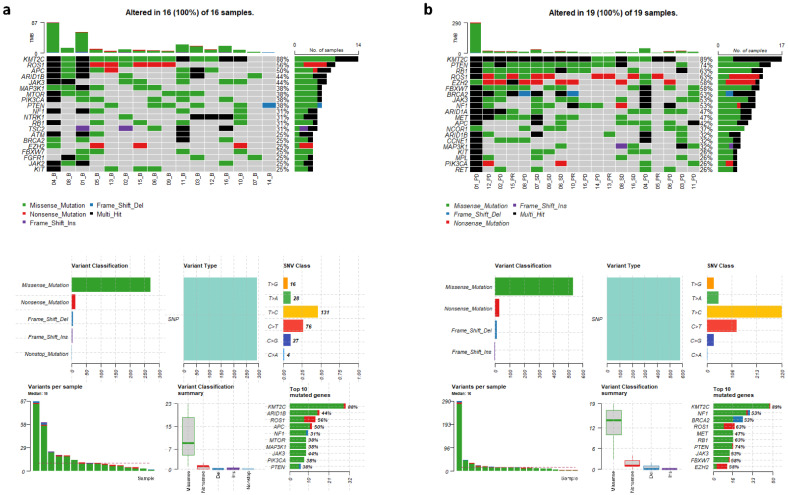
The top 20 most frequently mutated genes in individual metastatic breast cancer patients and summary of mutational features for blood samples collected at baseline (**a**) and follow-up (**b**). Note that three patients each had two different follow-up samples collected, accounting for the difference in number of baseline samples (16 in (**a**)) versus follow-up samples (19 in (**b**)).

In order to gain a global view of genomic aberrations and compare mutated genes identified at baseline and follow-up, 35 cfDNA samples underwent target sequencing at a mean depth of 2435× for captured regions. In the 16 baseline cfDNA samples, we identified 67 mutated genes in total and a median of 10 variants per sample, with missense mutations as the predominant mutation type and T > C substitutions as the major single nucleotide variant (SNV) class (Figure 1a). cfDNA samples from younger MBC patients (<54.7 years) had a significantly higher number of mutations (*p* = 0.035). Patients who were heavily treated (≥4 lines) or with IBC-tumor type also harbored more detectable mutations in the baseline samples than their counterparts, but the differences were not statistically significant (*p* = 0.308 and 0.358, respectively). Figure 1a shows the top 20 most commonly mutated genes in the 16 baseline cfDNA samples. Our results revealed that *KMT2C* was the most frequently mutated gene (88%), which is consistent with our recent report on ER^+^ IBC patients [24], followed by progressively less frequent mutations in *ROS1*, *APC*, *ARID1B*, *JAK3*, *MAP3K1*, *MTOR*, *PIK3CA*, and *PTEN*. Mutated *KMT2C* ranked at the top in both IBC (89%) and non-IBC (86%) baseline samples (*p* = 1.00), but the frequencies of other commonly mutated genes were quite different between the baseline samples collected from IBC and non-IBC patients. For example, *FBXW7* and *CDK6* were more frequently altered in IBC than in non-IBC (33% vs. 14%, *p* = 0.585; 22% vs. 14%, *p* = 1.00, respectively), whereas conversely, *PIK3CA* was more frequently altered in non-IBC than in IBC (57% vs. 22%, *p* = 0.302, Figure S1). We also observed co-occurrence of genomic alterations. For example, mutations in both *RB1* and *EZH2*, as well as mutations in both *ARID1B* and *FGFR1* were detected in the baseline samples from the same patients (Figure S2). 

Upon analysis of the 19 plasma samples collected at follow-up visits, we identified 87 mutated genes, including 20 mutated genes undetectable in the baseline samples. Moreover, we observed that the cfDNA samples at follow-up had more mutations than the baseline samples (median variants per sample 16 vs. 10, *p* = 0.260). Although *KMT2C* remained the gene with the highest mutation frequency in the follow-up samples, several genes in the cell cycle pathway, including *RB1* and *CCNE1*, displayed increased mutation frequencies in the samples collected at follow-up as compared to those collected at baseline (*RB1*, 63% vs. 31%, *p* = 0.060; *CCNE1*, 32% vs. 0%, *p* = 0.022, Figure 1b).

### 3.3. Identification of Significantly Mutated Genes at Progression by Paired Analyses

**Figure 2:**
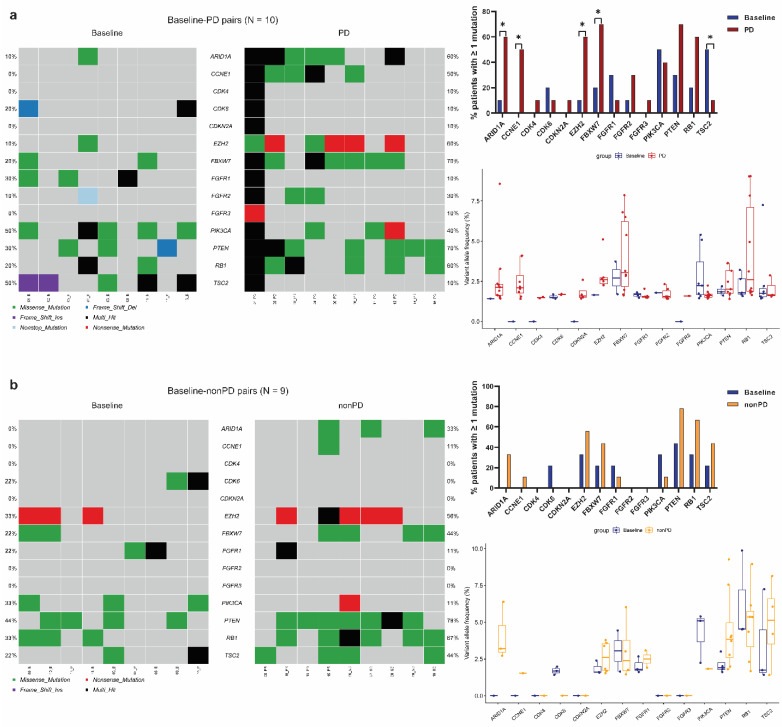
Differences in mutated gene frequencies and variant allele frequencies between samples collected at baseline and the corresponding paired samples collected at follow-up from each patient. Follow-up samples were categorized as PD or nonPD. Comparisons were conducted in baseline-PD pairs (**a**) and in baseline-nonPD pairs (**b**). PD: progressive disease. nonPD indicates without progression, including stable disease and partial response in this study. * represents *p* < 0.05.

Our unpaired analyses showed that cfDNA samples at follow-up had significantly increased mutation frequencies in genes such as *CCNE1*. To eliminate variations across subjects and identify more mutated genes that may potentially be involved in resistance to palbociclib-containing regimens, we further conducted paired analyses to compare frequencies of all the 87 mutated genes between paired baseline and follow-up cfDNA samples from the same patients. We found that PD samples contained significantly more detectable mutations than paired baseline samples in the genes *ARID1A* (60% vs. 10%, *p* = 0.025), *CCNE1* (50% vs. 0%, *p* < 0.001), *EZH2* (60% vs. 10%, *p* = 0.025), and *FBXW7* (70% vs. 20%, *p* = 0.025), whereas significantly fewer mutations were observed in *TSC2* in PD vs. baseline samples (10% vs. 50%, *p* = 0.046) (Figure 2a). Moreover, the same trend was observed when comparing median VAF of each mutated gene in the PD samples with corresponding baseline samples. In contrast, in the baseline-nonPD pairs (Figure 2b), although samples collected at follow-up had slightly increased mutation frequencies than their baseline samples regarding the aforementioned genes, none of the comparisons reached statistical significance. In addition to the results of five significantly altered genes (*ARID1A*, *CCNE1*, *EZH2*, *FBXW7*, and *TSC2*) identified in the present study, Figure 2 also shows the sequencing results of paired samples for the other nine mutated genes (*CDK4*, *CDK6*, *CDKN2A*, *FGFR1*, *FGFR2*, *FGFR3*, *PIK3CA*, *PTEN*, and *RB1*) which were associated with resistance to CDK4/6 inhibitors in previous clinical or preclinical studies of breast cancer [11,25,26,27]. In the PALOMA-3 trial [11], MBC patients had more detectable *RB1* mutations at the end of palbociclib treatment than paired baseline plasma samples (*p* = 0.041). In comparison, our paired analysis exhibited a nonsignificant increase in the frequency of mutated *RB1* in PD vs. baseline samples (*p* = 0.157). We did not obtain a significant result either when analyzing *FGFR1/2/3* gene mutations. The differences between our findings and others may be attributed to different study designs (observational study vs. clinical trial), patient characteristics (e.g., age, race, tumor stage, previous treatment), CDK4/6 inhibitor (palbociclib vs. ribociclib), sequencing approach (targeted vs. exome sequencing), and sample size.

### 3.4. Acquired Mutations in Cell Cycle Pathway Genes

**Figure 3:**
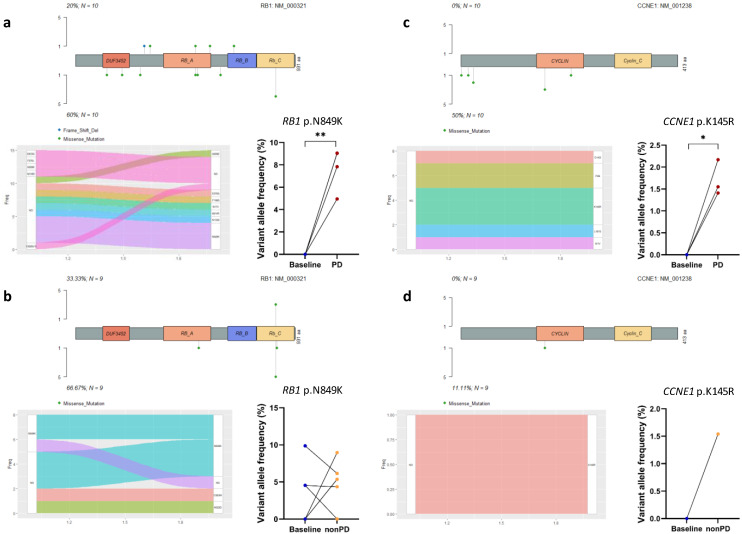
Changes in individual mutations in cell cycle pathway genes and changes in variant allele frequency of a given mutation from baseline to follow-up as determined by analyzing paired samples. (**a**,**b**) show the changes in *RB1* mutations in baseline-PD pairs or baseline-nonPD pairs. (**c**,**d**) show the changes in *CCNE1* mutations in baseline-PD pairs or baseline-nonPD pairs. PD: progressive disease; ND: not detected. * represents *p* < 0.05; ** represents *p* < 0.01.

The above paired analyses suggested a relationship between genomic variants and treatment failure. We then evaluated the alterations in mutated genes and recurrent mutations according to their involvement in signaling pathways. The cell cycle pathway is a key pathway in ER^+^ breast cancer and the target of CDK4/6 inhibitors [5]. The CDK4/6 complex acts as a checkpoint during the cell cycle transition from the cell growth (G1) phase to the DNA synthesis (S) phase, and CDK4/6 inhibition induces cell cycle arrest [5]. RB1 is the main target of the CDK4/6–cyclin D complex and plays a critical role in cell cycle regulation [26,27]. In this study, acquired *RB1* mutations were observed in six out of ten patients who developed progression (Figure 3a), but were only detected in four out of nine patients who did not progress (Figure 3b) (60% vs. 44%, *p* = 0.656), and of note, none of these four patients (No. 6, 7, 9, and 16) exhibited any treatment response (Table 1). We further compared the VAFs of one recurrent missense mutation in *RB1* that causes the amino acid substitution N849K in both baseline-PD pairs and baseline-nonPD pairs, and observed a significant increase in the VAFs for the PD samples (*p* = 0.004, Figure 3a), but not in the nonPD samples (*p* = 0.442, Figure 3b). *CCNE1* encodes cyclin E1, which has functions in cell cycle progression both in CDK-dependent and CDK-independent manners [28]. In the present study, we failed to detect any *CCNE1* mutations in the baseline samples; however, half of the PD patients (5/10 vs. 1/9 in nonPD patients, *p* = 0.141) acquired *CCNE1* mutations with dramatically increased VAFs (*p* = 0.018) after receiving palbociclib-containing regimens (Figure 3c,d). In addition, we only detected very low mutation frequencies in the cell cycle component genes *CDK4*, *CDK6*, and *CDKN2A* in both baseline and follow-up patient samples (Figure 2). Thus, our data not only confirmed acquired *RB1* mutations as previously reported [11,27], but also suggested novel acquired mutations in other cell cycle pathway genes, such as *CCNE1*, when MBC patients developed resistance to palbociclib-containing regimens. 

### 3.5. Acquired Mutations in Other Oncogenic Signaling Pathway Genes

**Figure 4:**
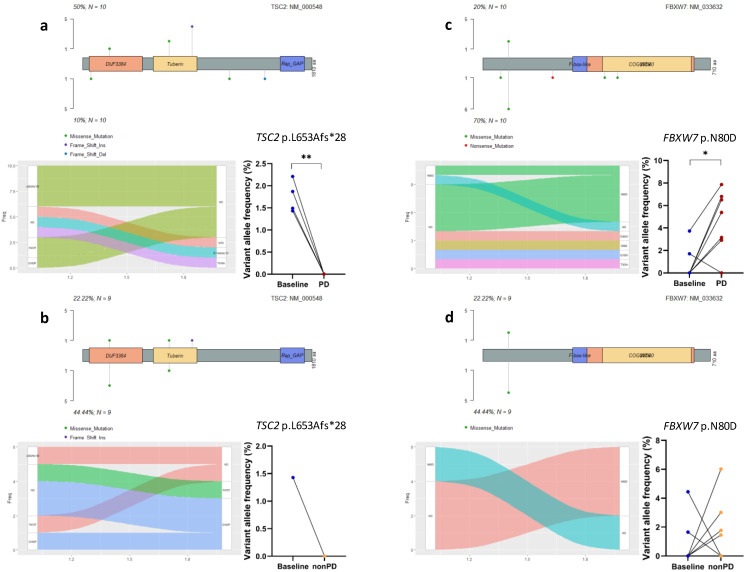
Changes in individual mutations in PI3K and Notch pathway genes and changes in variant allele frequency of a given mutation from baseline to follow-up as determined by analyzing paired samples. (**a**,**b**) show the changes in *TSC2* mutations in baseline-PD pairs or baseline-nonPD pairs. (**c**,**d**) show the changes in *FBXW7* mutations in baseline-PD pairs or baseline-nonPD pairs. PD: progressive disease; ND: not detected. * represents *p* < 0.05; ** represents *p* < 0.01.

Many CDK4/6 inhibitor resistance drivers can be broadly subdivided into two categories, including alterations in cell cycle mediators and activation of oncogenic signal transduction pathways [27]. After demonstrating that MBC patients acquired mutations in several genes in the cell cycle pathway, we then analyzed somatic alterations in other canonical signaling pathways such as phosphatidylinositol 3-kinase (PI3K), Notch, and receptor-tyrosine kinase (RTK)/RAS pathways, which are frequently detected in various cancers including MBC [29]. Aberrations in the PI3K pathway have been characterized predominantly as activating events in *PIK3CA* and inactivating events in *PTEN* [29]. In this study, we did not observe a significant difference in the frequencies of mutated *PIK3CA* or *PTEN* between paired baseline and follow-up samples (*p* = 0.564 and 0.103, respectively, Figure 2). Interestingly, we found that *TSC2*, another tumor suppressor in the PI3K pathway [29], exhibited the loss of mutations in five PD samples, but only in one nonPD (SD) sample (50% vs. 11%, *p* = 0.141). Figure 4a,b show an example of genomic variants in *TSC2*, a frameshift-inducing insertional alteration leading to the amino acid sequence change L653Afs*28, which was undetectable in the post-treatment samples. 

The Notch pathway is involved in embryological development and promotes proliferative signaling, and *FBXW7* is a negative regulator of the Notch pathway [29]. In this study, we found more patients with acquired *FBXW7* mutations in the PD samples (6/10) than in the nonPD samples (4/9, *p* = 0.656), as compared to corresponding baseline samples. The *FBXW7* N80D amino acid substitution was recurrently detected in these samples, exhibiting significantly increased VAFs in the PD samples when comparing to paired baseline samples (*p* = 0.012, Figure 4c), whereas the difference was not significant in the baseline versus nonPD pairs comparison (*p* = 0.522, Figure 4d). Intriguingly, more (4/7, 57%) non-IBC patients (No. 2, 4, 6, 12) acquired this *FBXW7* mutation in their PD samples than IBC patients (No. 8, 11%, *p* = 0.106), whereas two (No. 1, 10) out of three IBC patients with this mutation at baseline lost it after treatment. This result has never been reported by any of previous studies exploring molecular mechanisms of resistance to the CDK4/6 blockade.

Our study also identified significant genomic alterations in the genes *EZH2* and *ARID1A*, which are involved in epigenetic regulation and chromatin remodeling [30,31]. The majority (60%) of MBC patients acquired mutations in each of these two genes when they developed progression, and a similar trend was observed in VAF changes for two recurrent mutations, a stop-gain variant p.C510fs*0 in *EZH2* and a missense mutation p.Q199L in *ARID1A* (Figure S3). 

All these results suggested a relationship between genomic alterations in these oncogenic signaling pathway genes and treatment resistance.

### 3.6. Survival Analyses of Acquired Gene Mutations

**Figure 5:**
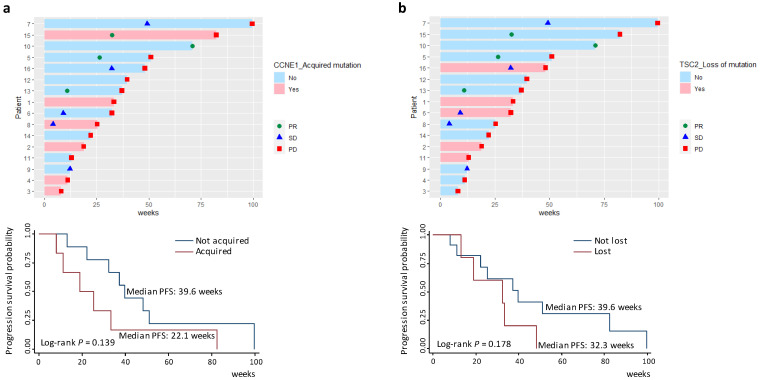
RECIST responses during follow-up for individual patients and survival difference between patients who acquired or did not acquire *CCNE1* mutations (**a**) and between patients who lost or did not lose *TSC2* mutations (**b**). RECIST: the Response Evaluation Criteria in Solid Tumors; PD: progressive disease; PR: partial response; SD: stable disease.

Owing to our above findings that at disease progression MBC patients acquired mutations in genes involved in the cell cycle pathway and other oncogenic singnaling pathways such as PI3K and Notch, we further conducted survival analyses to investigate the associations between these mutated genes and clinical outcome. Figure 5 depicts RECIST responses (see Materials and Methods) during follow-up for individual patients based on their mutational features of *CCNE1* and *TSC2*. As shown in Figure 5a, MBC patients who acquired *CCNE1* mutations experienced nonsignificantly reduced PFS compared to those who did not (median PFS 22.1 vs. 39.6 weeks, log-rank *p* = 0.139). The survival difference reached statistical significance (log-rank *p* = 0.049) when PD status was determined by the first imaging test after treatment initiation (Figure S4). On the contrary, patients with the loss of *TSC2* mutations in the follow-up samples tended to have nonsignificantly shorter PFS (median PFS 32.3 vs. 39.6 weeks, log-rank *p* = 0.178) (Figure 5b). In addition, survival difference between patients with or without mutation acquisition in the genes *RB1*, *FBXW7*, *EZH2*, or *ARID1A* (Figure S5) was not statistically significant, likely due to the small number of patients analyzed. Thus, our results indicated prognostic relevance of genomic alterations, especially the acqusition of *CCNE1* mutations. Future large studies are required to further clarify the associations of these genomic variants with patient outcomes.

## 4. Discussion

Palbociclib is the first-in-class CDK4/6 inhibitor approved for the treatment of patients with ER^+^/HER2^-^ advanced breast cancer. Although CDK4/6 inhibitors have changed clinical outcomes of ER^+^ MBC by prolonging PFS and delaying disease progression, the emergence of resistance remains inevitable in the metastatic context. Our current knowledge of the molecular mechanisms of resistance to CDK4/6 inhibitors is far from complete. To identify genomic aberrations associated with resistance to palbociclib, 35 cfDNA samples from 16 palbociclib-treated ER^+^ MBC patients were sequenced using a 91-gene targeted panel. Paired analyses of samples taken at baseline and follow-up from each patient revealed increased mutation frequencies in the genes *RB1*, *CCNE1*, *FBXW7*, *EZH2*, and *ARID1A*, and a reduced frequency of *TSC2* mutations when the patients developed progression. Moreover, the acquisition of *CCNE1* mutations and the loss of *TSC2* mutations conferred an unfavorable prognosis.

The dysregulation of the cyclin–CDK–Rb axis by upregulation of cyclin–CDK activity and/or abrogation of suppressors is a feature of many tumor types, including ER^+^ breast cancer [5]. The mechanism of actions for CDK4/6 inhibitors is centered on RB1, the product of the retinoblastoma tumor susceptibility gene *RB1* [26,27]. Preclinical evidence has suggested that alterations in RB1 or other cell cycle regulators such as amplification of the cyclin E gene, *CCNE1*, may confer resistance to CDK4/6 inhibitors [10,26,27,32]. The PALOMA-3 trial provided important clinical evidence regarding the involvement of acquired *RB1* mutations in the emergence of resistance to palbociclib in advanced ER^+^ breast cancer [11,33]. In line with this finding, our study analyzing paired pre- and post-treatment samples showed that the acquisition of *RB1* mutations, especially the *RB1* N849K substitution, was common in MBC patients who developed progression during study follow-up. 

O’Leary et al. recently reported that copy number loss of *RB1* and gain in *CCNE1* in baseline ctDNA were associated with worse prognosis [33]. Cyclin E is necessary for the formation of pre-replication complexes on DNA as cells re-enter the cell cycle after quiescence, and it also activates the CDK2 holoenzyme and phosphorylates many targets at the G1-to-S phase transition of the cell cycle, including the RB1 protein [28]. It was reported that palbociclib efficacy was lower in pre-treated MBC patients (PALOMA-3) with high *CCNE1* mRNA expression [34], but not in previously untreated patients (PALOMA-2) [12]. In the NeoPalAna trial that evaluated neoadjuvant palbociclib in the treatment of early-stage breast cancer, *CCNE1* expression was significantly elevated in the palbociclib-resistant group after two weeks of treatment [35]. *CCNE1* amplification was also noted among MBC patients with rapid progression on abemaciclib, another CDK4/6 inhibitor [36]. Nevertheless, clinical evidence regarding the role of *CCNE1* mutations in resistance to CDK4/6 inhibitors is still lacking. In our cohort of MBC patients, we found that a significantly higher proportion of patients who developed disease progression had acquired *CCNE1* mutations after receiving palbociclib, and we further showed that the acquisition of *CCNE1* mutations was associated with reduced PFS, highlighting the possibility that *CCNE1* may serve as a genomic marker for acquired resistance to palbociclib. Further studies are needed to confirm our findings and explore the potential crosstalk between *CCNE1* and other genes in cell cycle regulation during resistance to CDK4/6 inhibitors in ER^+^ breast cancer.

CDK4 and CDK6 become active when they form heterodimers with D-type cyclins, which are upregulated and post-translationally modified in response to mitogenic signaling by the PI3K/AKT/mTOR and RAS/MAPK signal transduction cascades [5]. In luminal breast cancers, the PI3K pathway is one of the most highly altered pathways, and is associated with *PIK3CA* mutations, loss of *PTEN*, and abnormal downstream protein phosphorylation [37,38]. Acquired *PIK3CA* mutations at the time of progression were reported by the PALOMA-3 trial, but the mutation acquisition detected in both treatment and placebo arms indicates a resistance mechanism not specific to palbociclib [11]. Costa et al. recently revealed *PTEN* loss as one of the mechanisms of acquired resistance to the CDK4/6 inhibitor ribociclib [39]. However, in our present study, the frequencies of mutated *PIK3CA* and *PTEN* were not significantly different between the paired samples. Instead, we found that a significantly higher percentage of PD patients lost *TSC2* mutations, and that patients with this mutation loss exhibited decreased PFS. CDK4/6 activates mTORC1 by binding and phosphorylating TSC2 [40]. Recent studies showed that pharmacological inhibition of CDK4/6 led to a rapid, TSC2-dependent reduction of mTORC1 activity in multiple cell lines including the breast cancer cell line MCF7 [40], whereas genetic depletion of *TSC2* in MCF7 cells resulted in sustained mTORC1 activity during palbociclib treatment and evoked a complete senescence response [41]. Consistent with these cell culture findings, our study now provides the first clinical data that implicate *TSC2* mutational features in the development of acquired resistance to palbociclib in human breast cancer patients. Currently, the mTOR inhibitor everolimus combined with exemestane is an approved line of therapy following progression on a CDK4/6 inhibitor [27]. The ongoing trials will further shed light on the treatment efficacy of the addition of a novel PI3K pathway inhibitor (e.g., BYLieve trial), after progression on CDK4/6 inhibitors [42]. 

The F-box and WD repeat-containing protein FBXW7 also plays an important role in the proteasomal degradation of proteins involved in the regulation of cell proliferation and survival, such as c-Myc and cyclin E, thereby inducing cell cycle exit [43]. The *FBXW7* mutation was shown to augment breast cancer cell proliferation in vitro by upregulating cyclin E [44]. Hence, perturbation of *FBXW7* expression is considered as one of the major causes of cancer development and progression [43,45]. Interestingly, a meta-analysis reported opposing effects of *FBXW7* mRNA expression on overall survival in different subtypes of breast cancer [45]. In the present study, although cfDNA samples obtained at the time of disease progression harbored more *FBXW7* mutations than the corresponding baseline samples, no associated positive or negative effect on survival was identified.

As a key regulator of ERα activity, disrupted KMT2C contributes to hormone-driven breast cancer proliferation [46]. *KMT2C* mutations are frequently detected in ER^+^ breast cancer and have been associated with shorter PFS on anti-estrogen therapy [46,47,48]. It is therefore not surprising that *KMT2C* was the most frequently mutated gene observed in our study of ER^+^ MBC patients with multiple lines of previous treatments. *KMT2C* encodes a histone methyltransferase [48], thus the high mutation frequency of *KMT2C* observed in this study suggests the role of epigenetic dysregulation in breast cancer. However, we did not notice a high proportion of patients who acquired *KMT2C* mutations after receiving palbociclib plus endocrine therapy, indicating that these *KMT2C* mutations are likely to play some other roles (e.g., chromosomal instability and DNA damage [49]) rather than the development of resistance to this therapeutic regimen. Intriguingly, PD samples acquired more mutations in *EZH2* and *ARID1A*, two other histone-modifying genes [50], although patient survival was not significantly different from those individuals who did not acquire these mutations, likely due to the small sample size. Epigenetic alterations are underexplored but emerging markers of treatment response to CDK4/6 inhibitors [26]. The results of the present study, although preliminary, point towards an interesting direction for future research. 

IBC is the most aggressive form of breast cancer. Since IBC patients comprised over half of our study cohort, we observed notable differences in mutation frequencies between IBC and non-IBC patients (e.g., *FBXW7*, *CDK6*, and *PIK3CA*). Currently, only a few studies have compared genomic profiles between IBC and non-IBC. Our results showed that *FBXW7* (Notch pathway gene) and *CDK6* (cell cycle pathway gene) were more frequently altered in IBC, whereas *PIK3CA* was more frequently altered in non-IBC, which was consistent with a recent publication. In a pooled analysis of next-generation sequencing data from 101 IBC and 2351 non-IBC tumors, Bertucci et al. demonstrated that the Notch and DNA repair pathways were more frequently targeted by genomic alterations in IBC than in non-IBC, whereas *PIK3CA* was the only gene that was more frequently altered in non-IBC (39.2% vs. 20.8% in IBC) [51]. Moreover, our data showed that IBC patients derived durable benefits from palbociclib plus endocrine therapy, although they had been pretreated with a significant number of therapy lines (Table 1). We recently reported that *FBXW7* mutations may be driver gene mutations for IBC by whole-exome sequencing of IBC tissue samples [24]. In the current study analyzing cfDNA sequencing data, we also found that *FBXW7* was more frequently altered in IBC than in non-IBC (33% vs. 14%) at baseline, and we further identified more non-IBC than IBC patients (57% vs. 11%) acquired a recurrent *FBXW7* mutation in their PD samples. Thus, the observed treatment benefit in IBC patients might be mechanistically related to the different genomic drivers and acquired mutations (e.g., *FBXW7*) when comparing IBC with non-IBC.

Our study, although informative, has several limitations. First, the number of patients analyzed was relatively small, and our findings are therefore preliminary and warrant further testing in prospective studies with a larger sample size. Second, matched germline DNAs from leukocytes were not sequenced in this study, and thus germline mutations were not fully excluded. Third, the majority of MBC patients received multiple lines of previous therapies, which may partially explain the high frequencies of mutated genes observed in this study. Previously treated patients, especially those being heavily treated, are more likely to acquire mutations under the selection pressure of systemic or targeted therapies. Thus, our current findings need to be confirmed and extended in future studies conducted in treatment-naïve MBC patients or within the first two lines of therapy. Fourth, somatic mutations have been linked to resistance to endocrine therapy. For example, Xu et al. recently identified a high frequency of *ARID1A* inactivating mutations in endocrine-resistant ER^+^ breast cancer [52]. However, due to the lack of a suitable comparison group composed of patients receiving only fulvestrant or letrozole, we cannot associate the genomic alterations identified in this study specifically to resistance to the CDK4/6 inhibitor, or to endocrine therapy, or to the combination of both treatments. Finally, the generalization of our findings may be restricted due to a high percentage of IBC patients included in this study. Data on CDK4/6 inhibitors are rarely reported in women with IBC because they are usually excluded from clinical trials. Our study subjects enrolled from an IBC-specific clinic; therefore, they provide a unique source for investigating treatment benefits and resistance in this underexplored population. 

## 5. Conclusions

Our results demonstrate that by targeted sequencing of paired cfDNA samples collected at baseline and follow-up, we have been able to detect genomic alterations after the initiation of palbociclib-containing regimens for the treatment of MBC. Our analyses reveal that the acquisition of *CCNE1* mutations and the loss of *TSC2* mutations confer an unfavorable prognosis, and suggest that these mutations may potentially serve as novel genomic biomarkers for treatment resistance. Future large-scale population studies and mechanism-focused research are needed to confirm the findings of this study and elucidate the underlying molecular mechanisms. Ultimately, such efforts may lead to the development of improved methods to predict treatment efficacy and clinical outcomes, as well as more effective targeted treatment approaches for the benefit of breast cancer patients.

## Figures and Tables

**Table 1:** Clinical characteristics of 16 MBC patients receiving palbociclib.

No	Age (yr)	Race	ER/PR /HER2	Tumor Differentiate	IBC	Lines of Previous Therapies *	Regimen	Number of Blood Samples		Response at EOT	TTP (wk)
								Baseline	F/U		
1	54.56	White	+/+/+	Poorly	Yes	6	Palbociclib + TDM-1	1	1 (PD)	PD	33.29
2	66.05	Black	+/+/−	Poorly	No	4	Palbociclib + letrozole	1	1 (PD)	PD	18.86
3	33.03	White	+/+/−	Poorly	Yes	0	Palbociclib + fulvestrant	1	1 (PD)	PD	8.00
4	42.79	White	+/+/−	Moderately	No	1	Palbociclib + fulvestrant	1	1 (PD)	PD	11.14
5	39.42	White	+/+/−	Poorly	No	2	Palbociclib + letrozole	1	1 (PR)	PD	51.00
6	58.14	White	+/+/−	Moderately	No	1	Palbociclib + fulvestrant	1	1 (SD), 1 (PD)	PD	32.29
7	66.25	White	+/+/−	Moderately	Yes	3	Palbociclib + fulvestrant	1	1 (SD)	PD	99.57
8	39.20	White	+/−/−	Poorly	Yes	8	Palbociclib + letrozole	1	1 (SD), 1 (PD)	PD	25.29
9	54.87	White	+/+/−	Unknown	Yes	1	Palbociclib + fulvestrant	1	1 (SD)	SD (Side effects)	(12.14)
10	32.56	Black	+/+/−	Poorly	Yes	1	Palbociclib + letrozole	1	1 (PR)	PR^#^ (LTFU)	(70.86)
11	51.12	White	+/−/−	Poorly	Yes	3	Palbociclib + letrozole	1	1 (PD)	PD	13.00
12	74.81	White	+/+/−	Moderately	No	4	Palbociclib + letrozole	1	1 (PD)	PD	39.57
13	45.15	White	+/+/−	Poorly	Yes	1	Palbociclib + fulvestrant	1	1 (PR)	PD	37.14
14	64.12	White	+/−/−	Moderately	No	4	Palbociclib + fulvestrant	1	1 (PD)	PD	22.14
15	58.62	White	+/+/−	Poorly	Yes	1	Palbociclib + fulvestrant	1	1 (PR)	PD	82.29
16	63.78	White	+/−/−	Moderately	No	0	Palbociclib + letrozole	1	1 (SD), 1 (PD)	PD	48.14

MBC: metastatic breast cancer; yr: year; ER: estrogen receptor; PR: progesterone receptor; HER2: human epidermal growth factor receptor 2; IBC: inflammatory breast cancer; F/U: follow-up; PD: progressive disease; PR^#^: partial response; SD: stable disease; EOT: end of treatment; LTFU: loss to follow-up; TTP: time to progression; wk: week. * Lines of previous therapies from MBC diagnosis to baseline sample collection.

## Data Availability

The data that support the findings of this study are available from the corresponding author upon reasonable request.

## Supplementary Materials

The following supporting information can be downloaded at: https://www.mdpi.com/article/10.3390/cancers14122872/s1, Figure S1: The top 20 most frequently mutated genes in blood samples collected at baseline for individual patients with IBC or non-IBC; Figure S2: Co-occurrence and mutual exclusivity of mutated genes detected in baseline blood samples; Figure S3: Changes in individual mutations in genes *EZH2* and *ARID1A* that are involved in epigenetic regulation and changes in variant allele frequency of a given mutation from baseline to follow-up as determined by analyzing paired samples; Figure S4: Kaplan–Meier curve showing survival difference between patients who acquired or did not acquire *CCNE1* mutations; Figure S5: RECIST responses during follow-up for individual patients and survival difference between patients who acquired or did not acquire mutations in genes *RB1*, *FBXW7*, *EZH2*, and *ARID1A*; Table S1: The panel of 91 breast-cancer-related genes.
