# Supplementary material for: Genomic Aberrations in Circulating Tumor DNAs from Palbociclib-Treated Metastatic Breast Cancer Patients Reveal a Novel Resistance Mechanism
Source: Cancers (Basel). 2022 Jun 10;14(12):2872. doi: 10.3390/cancers14122872 (PMC9221535; doi:10.3390/cancers14122872)
Supplement: Supplementary file 1 [file cancers-14-02872-s001.zip › cancers-1728396-supplementary.pdf]

Table S1. The panel of 91 breast cancer-related genes.

Gene							
AKT1	*CCND1*	*ESR1*	*HNF1A*	*MAP2K4*	*NF2*	*PTPN11*	*TBX3*
ALK	*CCND2*	*EZH2*	*HRAS*	*MAP3K1*	*NFE2L2*	*RAF1*	*TERT*
APC	*CCND3*	*FBXW7*	*IDH1*	*MAPK1*	*NOTCH1*	*RB1*	*TP53*
AR	*CCNE1*	*FGFR1*	*IDH2*	*MAPK3*	*NPM1*	*RET*	*TSC1*
ARAF	*CDH1*	*FGFR2*	*IGF1R*	*MDM2*	*NRAS*	*RHEB*	*TSC2*
ARID1A	*CDK4*	*FGFR3*	*JAK2*	*MET*	*NTRK1*	*RHOA*	*VHL*
ARID1B	*CDK6*	*FOXA1*	*JAK3*	*MLH1*	*NTRK3*	*RIT1*	*ZNF21*
ATM	*CDKN2A*	*GATA3*	*KIT*	*MPL*	*PDGFRA*	*ROS1*	
BRAF	*CTNNB1*	*GNA11*	*KMT2C*	*MTOR*	*PIK3CA*	*SMAD4*	
BRCA1	*DDR2*	*GNAQ*	*KRAS*	*MYC*	*PIK3R1*	*SMO*	
BRCA2	*EGFR*	*GNAS*	*MAP2K1*	*NCOR1*	*PTCH1*	*SOS*	
CBFB	*ERBB2*	*GRB2*	*MAP2K2*	*NF1*	*PTEN*	*STK11*	

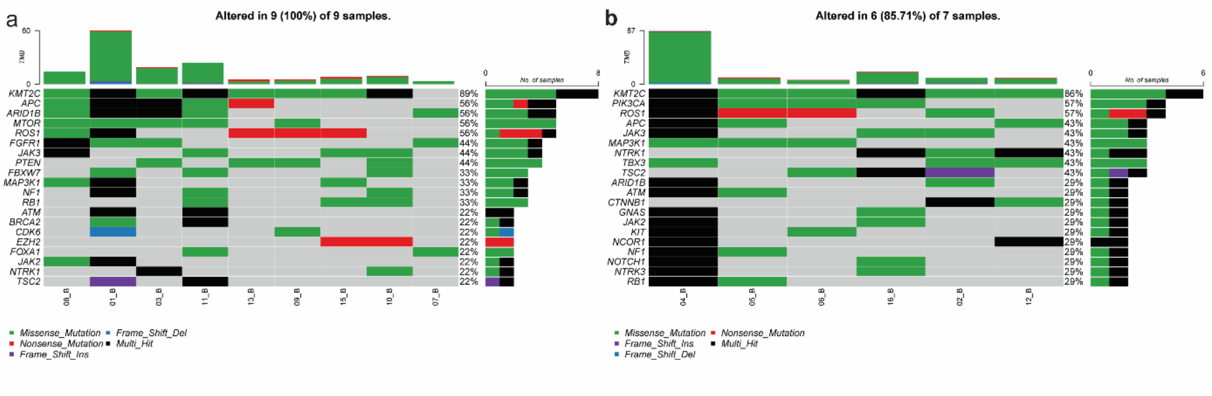

Figure S1. The top 20 most frequently mutated genes in blood samples collected at baseline for individual patients with IBC (**a**) or non-IBC (**b**). IBC: inflammatory breast cancer.

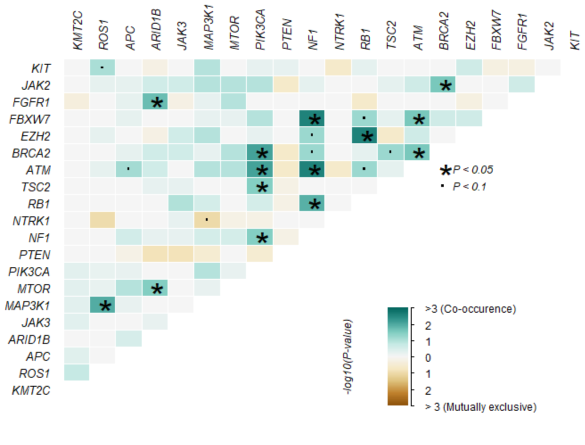

Figure S2. Co-occurrence and mutual exclusivity of mutated genes detected in baseline blood samples. Matrix depicting the degrees of co-occurrence (green scale) and mutual exclusivity (orange scale) of all possible pairwise combinations of any two mutated genes across a dataset of 20 analyzed genes.

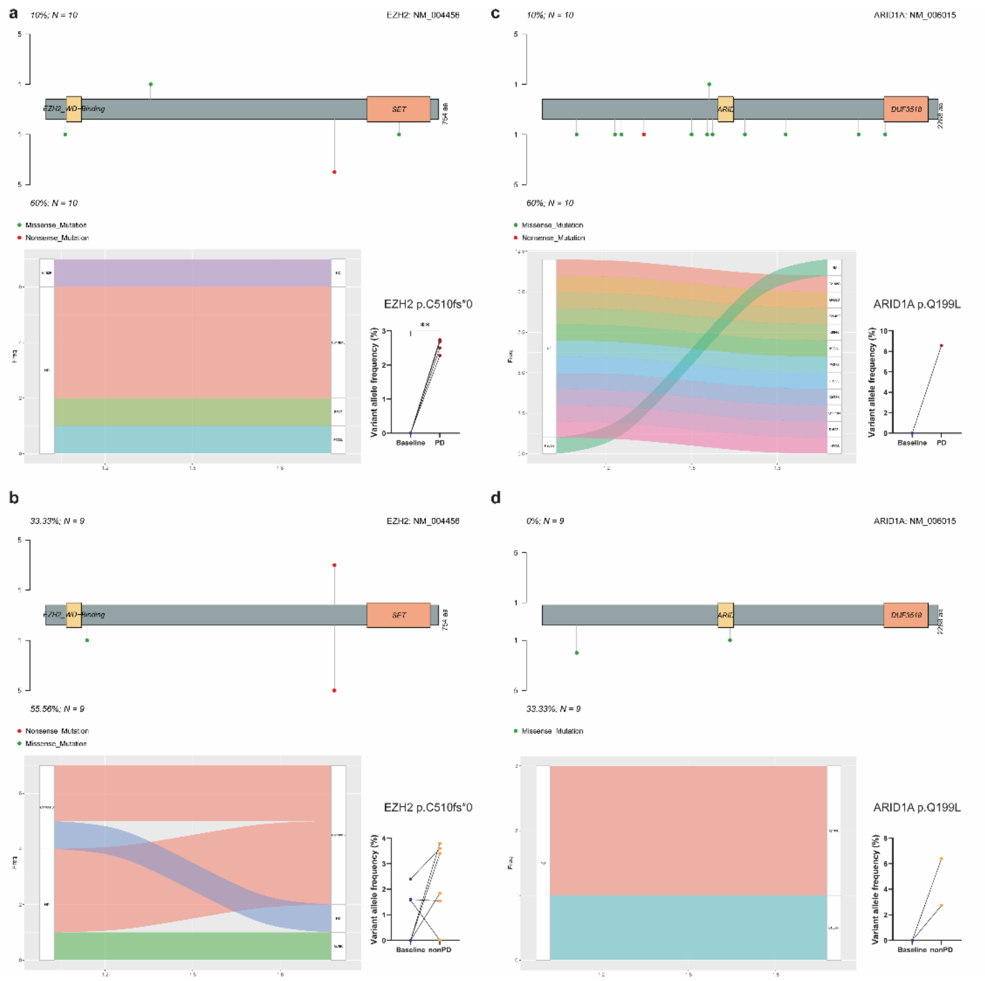

Figure S3. Changes in individual mutations in genes involved in epigenetic regulation and changes in variant allele frequency of a given mutation from baseline to follow-up as determined by analyzing paired samples. (**a**) and (**b**) show the changes in *EZH2* mutations in baseline-PD pairs or baseline-nonPD pairs. (**c**) and (**d**) show the changes in *ARID1A* mutations in baseline-PD pairs or baseline-nonPD pairs. PD: progressive disease; ND: not detected. ** represents $P < 0.01$.

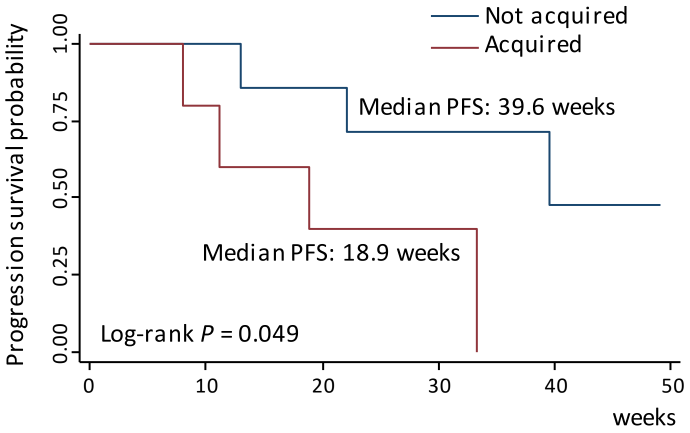

Figure S4. Kaplan-Meier curve showing survival difference between patients who acquired or did not acquire *CCNE1* mutations. PD status was determined by the first imaging test after treatment initiation. PD: progressive disease.

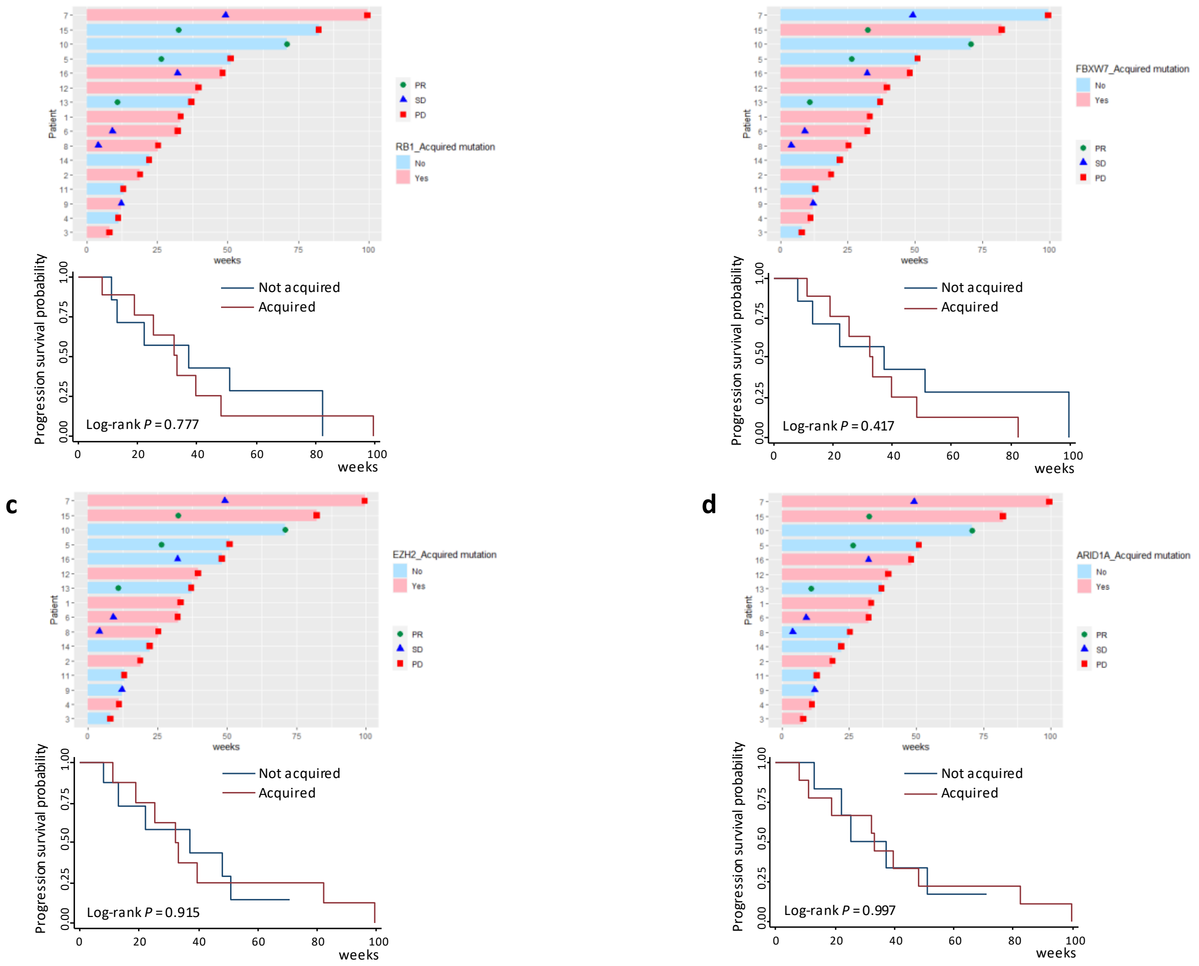

Figure S5. RECIST responses during follow-up for individual patients and survival difference between patients who acquired or did not acquire mutations in genes *RB1* (**a**), *FBXW7* (**b**), *EZH2* (**c**), and *ARID1A* (**d**). RECIST: the Response Evaluation Criteria in Solid Tumors; PD: progressive disease; PR: partial response; SD: stable disease.
